# Nilotinib revisited in GIST: salvage use in patients with severe imatinib toxicity

**DOI:** 10.1093/oncolo/oyag345

**Published:** 2026-09-03

**Authors:** Johanna Falkenhorst, Peter Hohenberger, Giorgia Mancino, Andrea Scrima, Franka Menge, Benjamin S Fletcher, Carina Reiff, Valerie Lange, Rainer Hamacher, Stefanie Bertram, Fiona M Rau, Yasmin Zaun, Moritz Kaths, Jürgen Treckmann, Jens Jakob, Thomas Mühlenberg, Susanne Grunewald, Dawid Krzeciesa, Daniel Rauh, Sebastian Bauer

**Affiliations:** Department of Medical Oncology and Sarcoma Center, West German Cancer Center, University Duisburg-Essen, Medical School, Essen, 45122, Germany; DKTK Partner Site Essen, German Cancer Consortium (DKTK), Heidelberg, 69120, Germany; National Center for Tumor Diseases (NCT) West, Essen, 45122, Germany; Division of Surgical Oncology, Medical Faculty Mannheim of University of Heidelberg; Department of Chemistry and Chemical Biology, TU Dortmund University, Dortmund, 44227, Germany; Drug Discovery Hub Dortmund (DDHD) am Zentrum für Integrierte Wirkstoffforschung (ZIW), Dortmund, 44227,Germany; Department of Chemistry and Chemical Biology, TU Dortmund University, Dortmund, 44227, Germany; Drug Discovery Hub Dortmund (DDHD) am Zentrum für Integrierte Wirkstoffforschung (ZIW), Dortmund, 44227,Germany; Sarcoma Unit, Department of Surgery, Medical Faculty Mannheim, Heidelberg University, Mannheim, 68167, Germany; Department of Medical Oncology and Sarcoma Center, West German Cancer Center, University Duisburg-Essen, Medical School, Essen, 45122, Germany; DKTK Partner Site Essen, German Cancer Consortium (DKTK), Heidelberg, 69120, Germany; Department of Medical Oncology and Sarcoma Center, West German Cancer Center, University Duisburg-Essen, Medical School, Essen, 45122, Germany; DKTK Partner Site Essen, German Cancer Consortium (DKTK), Heidelberg, 69120, Germany; Department of Medical Oncology and Sarcoma Center, West German Cancer Center, University Duisburg-Essen, Medical School, Essen, 45122, Germany; DKTK Partner Site Essen, German Cancer Consortium (DKTK), Heidelberg, 69120, Germany; National Center for Tumor Diseases (NCT) West, Essen, 45122, Germany; Department of Medical Oncology and Sarcoma Center, West German Cancer Center, University Duisburg-Essen, Medical School, Essen, 45122, Germany; DKTK Partner Site Essen, German Cancer Consortium (DKTK), Heidelberg, 69120, Germany; National Center for Tumor Diseases (NCT) West, Essen, 45122, Germany; DKTK Partner Site Essen, German Cancer Consortium (DKTK), Heidelberg, 69120, Germany; Department of Pathology, University Hospital Essen, Essen, 45122, Germany; DKTK Partner Site Essen, German Cancer Consortium (DKTK), Heidelberg, 69120, Germany; Department of Pathology, University Hospital Essen, Essen, 45122, Germany; Department of Medical Oncology and Sarcoma Center, West German Cancer Center, University Duisburg-Essen, Medical School, Essen, 45122, Germany; DKTK Partner Site Essen, German Cancer Consortium (DKTK), Heidelberg, 69120, Germany; National Center for Tumor Diseases (NCT) West, Essen, 45122, Germany; DKTK Partner Site Essen, German Cancer Consortium (DKTK), Heidelberg, 69120, Germany; Department of General, Visceral, Vascular and Transplantation Surgery, University Hospital Essen, Essen, 45122, Germany; DKTK Partner Site Essen, German Cancer Consortium (DKTK), Heidelberg, 69120, Germany; Department of General, Visceral, Vascular and Transplantation Surgery, University Hospital Essen, Essen, 45122, Germany; Division of Surgical Oncology, Medical Faculty Mannheim of University of Heidelberg; Department of Medical Oncology and Sarcoma Center, West German Cancer Center, University Duisburg-Essen, Medical School, Essen, 45122, Germany; DKTK Partner Site Essen, German Cancer Consortium (DKTK), Heidelberg, 69120, Germany; National Center for Tumor Diseases (NCT) West, Essen, 45122, Germany; Department of Medical Oncology and Sarcoma Center, West German Cancer Center, University Duisburg-Essen, Medical School, Essen, 45122, Germany; DKTK Partner Site Essen, German Cancer Consortium (DKTK), Heidelberg, 69120, Germany; National Center for Tumor Diseases (NCT) West, Essen, 45122, Germany; Department of Medical Oncology and Sarcoma Center, West German Cancer Center, University Duisburg-Essen, Medical School, Essen, 45122, Germany; DKTK Partner Site Essen, German Cancer Consortium (DKTK), Heidelberg, 69120, Germany; National Center for Tumor Diseases (NCT) West, Essen, 45122, Germany; Department of Chemistry and Chemical Biology, TU Dortmund University, Dortmund, 44227, Germany; Drug Discovery Hub Dortmund (DDHD) am Zentrum für Integrierte Wirkstoffforschung (ZIW), Dortmund, 44227,Germany; Department of Medical Oncology and Sarcoma Center, West German Cancer Center, University Duisburg-Essen, Medical School, Essen, 45122, Germany; DKTK Partner Site Essen, German Cancer Consortium (DKTK), Heidelberg, 69120, Germany; National Center for Tumor Diseases (NCT) West, Essen, 45122, Germany

**Keywords:** GIST, imatinib toxicity, nilotinib, salvage treatment, resistance

## Abstract

**Background:**

Severe imatinib toxicity is a rare, but relevant event in GIST patients. Other approved treatments were not evaluated within a first-line setting and exhibit severe adverse events that limit QoL. Nilotinib was evaluated within this setting in GIST with a safety profile comparable to imatinib and effectiveness within *KIT*-exon-11-mutant GIST subgroup (ENESTg1).

**Materials, patients, and methods:**

This is a retrospective case series reporting the outcome and side effects of 20 patients treated with nilotinib following severe imatinib toxicity. Nilotinib efficacy was tested in GIST cell lines carrying common primary and resistance mutations in *KIT*. Inhibitory profile of nilotinib was characterized using in silico modeling.

**Results:**

In our retrospective analysis of 1263 patients, 7.3% of patients discontinued imatinib due to toxicity. In 20 patients who received nilotinib, reasons were skin (*n* = 11) and liver (*n* = 4) toxicity, followed by cardiac (*n* = 2) and gastrointestinal toxicities (*n* = 2). No cross-toxicities were observed with nilotinib treatment. Nilotinib was effective after imatinib intolerance. A secondary KIT Exon 9 mutation was found in a progressing lesion. In vitro viability assays showed effectiveness in *KIT*-Exon-11-mutant cell lines, but not in Exon 9 or imatinib-resistant mutations in doses deemed clinically achievable. In silico modeling revealed steric hindrance by Exon 9 or imatinib resistance mutations.

**Conclusion:**

Nilotinib exhibits no cross toxicity with imatinib and represents an important alternative in imatinib-sensitive, *KIT*-exon-11-mutant GIST given its excellent toxicity profile. There is no biological rationale for the use of nilotinib in imatinib-resistant GIST with secondary KIT mutations.

Implications for practice Severe imatinib toxicity is a rare event in GIST patients. Of note, the switch to second-line sunitinib is currently the only approved treatment option. Sunitinib is associated with more frequent and more severe side effects which, unlike imatinib, may increase over time. Nilotinib may be a well-tolerated, efficient treatment option in a nonresistant disease scenario as cross toxicities are rare. With the loss of patent protection, nilotinib represents a highly affordable alternative in imatinib-sensitive *KIT*-exon-11-mutant GIST.

## Introduction

Gastrointestinal stromal tumors (GIST) are characterized by activating mutations in *KIT* or *Platelet-derived growth factor receptor alpha (PDGFRA)*.^1^^,^^2^ Patients can be successfully treated with tyrosine kinase inhibitors such as imatinib and metastatic GIST require life-long therapy.^3^ With a median age of 60 years,^4^ many patients are highly active at the time of diagnosis and minimizing side effects is of high importance. Most patients eventually progress on imatinib due to acquired *KIT* mutations mainly found within the kinase domain of KIT.^5^ Next line treatments with TKIs inhibit subsets of these mutations, but yield shorter progression free survival (5-7 months) than imatinib (18-24 months).^6^^,^^7^ More importantly, salvage drugs, particularly sunitinib and regorafenib represent multi-tyrosine kinase inhibitors that were primarily developed as inhibitors of angiogenesis for the treatment of renal and colorectal cancer. The side effect profile of these drugs is interfering with activities of daily living to a much greater extent than imatinib, particularly due to skin and gastrointestinal toxicity. While approved salvage drugs for the treatment of GIST are very likely to control primary metastatic disease as good as imatinib, the impact on the quality of life can be dramatic. Even mild stomatitis (°I Common Terminology Criteria for Adverse Events, CTCAE), which is experienced by many patients receiving sunitinib, may impact every meal, interfere with tasting or the general enjoyment of food. Hand-foot-syndrome, which is very common in patients with regorafenib, is perceived as a great impediment to mobility or patient’s ability to exercise. Ripretinib has a more favorable toxicity profile than sunitinib, as was evidenced in the INTRIGUE trial, but still, side effects are more common than with imatinib.^8–10^ Most of these side effects are absent in patients receiving imatinib, which is why alternatives for imatinib with little toxicity are so important, particularly as some patients stay on their first-line treatment for sometimes many years, even indefinitely.

Severe imatinib toxicity is fortunately rarely observed. The most common adverse events of grade 3 or higher were cytopenias, pain and fatigue, followed by edema, rash, liver toxicity, and dyspnea. However, all these events occurred in 2-10% and only 26% of 470 treated patients experienced any grade 3 side effects.^11^^,^^12^ In this case, the only in-label option for patients is to switch to the approved second line treatment sunitinib. Nilotinib, a second-generation tyrosine kinase inhibitor (TKI) has been developed and approved for the treatment of BCR::ABL1 positive chronic myelogenous leukemia (CML) in adults in first-line and for adults and children after resistance or intolerability to imatinib or any other first-line drug. Nilotinib was tested in GIST vs imatinib in a first-line setting (Phase 3 ENESTg1 trial), powered for superiority testing. Within the total cohort, nilotinib was inferior with a median PFS of 25.9 vs 29.7 months. However, the data strongly suggests similar effectivity in *KIT*-exon-11-mutant patients while being inactive in *KIT*-exon-9-mutant patients. Both drugs were well tolerated but differed slightly in their toxicity profile. Treatment had to be discontinued due to side effects in 5% (17/320) of patients after imatinib and 8% (26/324) after nilotinib treatment. Most common reasons were hyperbilirubinemia and vomiting in the imatinib group and anemia and neutropenia within the nilotinib group.^13^ Clinical trials investigating the use of nilotinib in later treatment lines showed a median PFS of 2 to 3.7 months.^14–16^ A phase 3 trial showed that nilotinib treatment after failure of imatinib and sunitinib was not superior to best supportive care or imatinib/sunitinib rechallenge.^17^

Notably, nilotinib is infrequently used off-label after approval by health insurances in patients displaying severe imatinib-associated toxicity. However, very little data is available on cross-toxicity for patients with GIST who have switched to nilotinib for imatinib intolerance. In addition, nilotinib had initially been developed as an improved version of imatinib but the potential improvement over imatinib has not been investigated in comprehensive models of *KIT* mutations. Mechanisms of resistance to nilotinib are not well described. We aimed to summarize clinical experience with nilotinib in imatinib-intolerant patients at our centers and to revisit nilotinib as a KIT-inhibitory drug in GIST using state-of-the-art in vitro models of GIST.

## Material and methods

### Characteristics of this study

This was a 2-center retrospective cohort study. The study was conducted in compliance with the declaration of Helsinki and was approved by the Ethics Committees of the University Duisburg-Essen, Germany (15-6339-BO) and the Medical Ethics Commission II, Medical Faculty Mannheim, University of Heidelberg (no. 2023-857).

The institutional GIST databases of the Sarcoma Center at the Essen University Hospital (*n* = 771) and the Sarcoma Center at Mannheim University Medicine (*n* = 595) were queried for patients with imatinib toxicity and consecutive nilotinib treatment. Patient data were obtained from the electronic hospital information system (HIS) of the 2 hospitals (Consort Diagram, Figure S1).

Descriptive statistics were obtained using Excel (Microsoft).

### Cell lines, cell culture, and viability assay

Cell lines were engineered and cultured as described previously.^18^ Viability assays and analyses were conducted as described before.^18^^,^^19^ Briefly, Sulforhodamine B (SRB) assays were conducted as described by Skehan et al.,^20^ the calculation of GR50 values and curve fitting was performed using the R-package Grmetrics,^21^ using the mean of >2 independent experiments.

### Structure-based modeling

In the absence of crystallographic data for the KIT-nilotinib complex, the co-crystal structures of ABL1 in complex with nilotinib (PDB ID 3CS9^22^) and imatinib (PDB ID 2HYY^23^) were superimposed with KIT-WT in active (PDB ID 1PKG^24^) and inactive (PDB ID 1T45 and 1T46^25^) conformation and with KIT-D816V (PDB ID 7KHK^26^) using PyMOL (The PyMOL Molecular Graphics System, Version 2.5.5 Schrödinger, LLC). PyMOL was also used to introduce the T670I and V654A point mutations (if not present in the experimental structures) and to generate the corresponding Figure. Pharmacophore models and interactions of imatinib and nilotinib in KIT and in ABL1 were generated using LigandScout^27^ to generate Figure S2.

## Results

### Imatinib toxicity leading to discontinuation of treatment is a rare event in clinical practice

The institutional GIST databases of Essen and Mannheim were queried to identify patients that had to discontinue imatinib treatment due to toxicity. Nine out of 144 patients quit neoadjuvant treatment (6.2%) and 33/313 withdrew from adjuvant treatment (10.5%). Within the palliative setting, 40/643 (6.2%) of patients treated with imatinib 400 mg qd and 11/163 (6.7%) treated with 400 mg bid discontinued treatment because of significant side effects (Table 1).

**Table 1.** Patients with treatment discontinuation due to imatinib toxicity.

Type of therapy	Total	Discontinuation (toxicity)	
	*N*	*N*	Percentage
**Imatinib 400 mg qd**			
** Neoadjuvant**	144	9	6.2%
** Adjuvant**	313	33	10.5%
** Palliative**	643	40	6.2%
**Imatinib 400 mg bid**			
** Palliative**	163	11	6.7%

### Nilotinib cohort: patient characteristics

This was a bi-centric retrospective analysis of patients treated with nilotinib after experiencing imatinib side effects leading to discontinuation of treatment at 2 high-volume sarcoma centers. We identified a total of 20 patients of which four patients had sunitinib treatment between imatinib and nilotinib treatment. Patients had a median age of 67 years at imatinib start and were predominantly female (female: 14/20). The primary tumor was located within the small intestine in 9 and within the stomach in 6 cases, followed by duodenum and rectum (*n* = 2) and unknown origin (*n* = 1). Metastases were located within peritoneum (*n* = 6), liver (*n* = 3) or both (*n* = 4). Two patients had a local recurrence of rectal GIST. Patients received imatinib in a perioperative setting in 7 and within a palliative setting in 13 cases (Table 2). Nilotinib was administered after approval of cost coverage by respective health insurance companies.

**Table 2.** Patient characteristics.

Variable	
**Age (median, range)**	
** At diagnosis**	64 y (34-77 y)
** At start of imatinib treatment**	67 y (51-77 y)
**Gender**	
** Male**	6 (30%)
** Female**	14 (70%)
**Primary tumor location**	
** Small intestine**	9 (45%)
** Stomach**	6 (30%)
** Duodenum**	2 (10%)
** Rectum**	2 (10%)
** Unknown**	1 (5%)
**Disease status at treatment start**	
** Localized disease**	7 (35%)
** Metastatic disease**	13 (65%)
**Metastasis/relapse location**	
** Liver**	3 (15%)
** Peritoneum**	6 (30%)
** Liver and peritoneum**	4 (20%)
** Local relapse**	2 (5%)
**Mutational status**	
** *KIT* Exon 11**	18 (90%)
** *KIT* Exon 10**	1 (5%)
** *KIT/PDGFRA* wildtype**	1 (5%)
**Imatinib treatment**	
** Neoadjuvant/perioperative**	5 (25%)
** Adjuvant**	2 (10%)
** Metastatic setting**	13 (65%)
**Imatinib toxicity**	
** Skin**	11 (55%)
** Liver**	5 (25%)
** Nausea**	3 (15%)
** Gastrointestinal/diarrhea**	2 (10%)
** Cardiac**	2 (10%)

### Imatinib/nilotinib cross-toxicities are rare events

In this cohort, imatinib was administered 400 mg once daily. All patients discontinued imatinib following intolerable side effects without evidence of progressive disease. The median duration of imatinib treatment until withdrawal due to toxicity was 18 weeks (range 4-69 weeks). In 17/20 cases, the toxicity occurred within the first 6 months. The most frequently observed side effects to imatinib were skin and liver-related toxicities (*n* = 11/20 and *n* = 5/20, respectively) followed by nausea (*n* = 3/20) and cardiac decompensation (*n* = 2/20). One patient experienced severe diarrhea, esophagitis and a hydropic decompensation with combined heart and liver toxicity, respectively. The side effects leading to imatinib discontinuation were rated CTCAE grade 3 or four except for one case of grade 2 skin toxicity (Pat. 2). Imatinib was discontinued in most patients (16/20) and the following treatment was started after improvement of side effects to grade one or two. Nilotinib was administered at doses from 200-400mg bid. The median treatment duration was 65.5 weeks (range 9-386 weeks). Adverse events observed during nilotinib treatment were reported in 12/20 patients. Three patients reported cardiovascular events (cerebellar infarction G3, hypertension G2, QTc prolongation G1), four patients reported gastrointestinal toxicities G1-2, 5 patients with skin toxicities and/or alopecia G1-2, fatigue (*n* = 2 G1-2) and elevated transaminases (G2-3, Table 3).

**Table 3.** Imatinib- and nilotinib-associated toxicities.

Pt	Sex	Imatinib				Nilotinib					
		Dose	Duration (w)	Toxicity	Grade	Dose	Duration (w)	DOR (w)	Best response	Toxicity	Grade
**1**	M	400mg qd	18	Skin	4	400mg bid	256		SD	Cerebellar infarction	3
**2**	F	400mg qd	15	Skin HFS	2	400mg bid	164		SD	None	
**3**	F	400mg qd	13	Skin Edema	3	400mg bid	335	36	NED	None	
**4^a^**	F	400mg qd	24	Skin	3-4	400mg bid	65	9	SD	Diarrhea Skin	1
**5**	F	400mg qd	30	Skin Nausea Edema	3	400mg bid	358+		PR	None	
**6**	F	400mg qd	18	Skin Nausea Diarrhea	3	400mg bid	26		PR	Skin Alopecia	1 2
**7**	F	400mg qd	31	Skin HFS Stomatitis	3	400mg bid	143		NED	Diarrhea	2
**8**	F	400mg qd	4	Skin	3	200mg bid	17		MR	Dumping Fatigue	1
**9**	M	400mg qd	23	Skin	3	400mg bid	66		PR	None	
**10**	F	400mg qd	8	Skin	3	400mg bid	235		PR	None	
**11**	M	400mg qd	8	Cardiac	3	400mg bid	36		PR	Fatigue	2
**12**	M	400mg qd	4	Liver	4	200mg bid	368	22	NED	None	
**13^a^**	F	400mg qd	26	Liver	3-4	400mg bid	43		SD	Transaminases elevated Pruritus	1 2
**14**	M	400mg qd	17	Liver	4	300mg bid	128+	128+	PR	Eczema Urge to urinate	1 2
**15**	F	400mg qd	22	Liver	unk	400mg bid	26		NED	Diarrhea Mucositis	2 2
**16^a^**	F	400mg qd	9	Diarrhea	3	400mg bid	43+	6	SD	Paresthesia QTc prolongation	1 1
**17^a^**	F	400mg qd	24	Nausea Leukopenia	unk	400mg bid	48		PR	None	
**18**	M	400mg qd	69	Esophagitis	3	300mg bid	152	152	SD	Hypertension	2
**19**	F	400mg qd	13	Cardiac	3	300mg bid	31	31	SD	None	
**20**	F	400mg qd	22	Skin	3	400mg bid	9		PR	Alopecia Transaminases elevated	1 3

Abbreviations: +, treatment ongoing; F, female; HFS, hand-foot-syndrome; M, male; none, no significant toxicity reported; Pt., patient; Tx, treatment; unk, unknown; w, weeks.

a Sunitinib treatment between imatinib and nilotinib treatment.

### Skin toxicities

Skin toxicity was the reason for imatinib discontinuation in 11/20 patients. In patients for which detailed information was accessible (4/10), acneiform rash, erythema, stomatitis and/or hand-foot-syndrome was reported. Patients were treated with topical and/or systemic steroids.

### Liver toxicities

Four patients experienced isolated asymptomatic grade 3-4 liver function test elevations (as measured by GOT, GPT, and bilirubin in serum). Liver toxicity as manifested by a grade 4 increase of transaminases and concomitant lactate dehydrogenase and/or bilirubin increase and/or alkaline phosphatase increase, led to treatment interruptions until reconstitution. In those cases where laboratory values were available hepatocellular injury was found (*R*-values >5). The criteria for Hy’s law were not met. Patient 13 received a liver biopsy that revealed portal accentuated inflammation with lobular single and small group necrosis partly in resorption, slight diffuse steatosis and increased portal and periportal fibrosis with initial ductular reaction; consistent with drug-induced liver injury.

### Cardiac toxicities

Two patients (Pt. 11 and 19) developed cardiac toxicities. Both had a hydropic decompensation leading to ICU admission in one case. Cardiac failure and consecutive liver enzyme elevation was diagnosed. Imatinib treatment was withdrawn. After recompensation nilotinib was administered without recurrence of cardiac toxicities.

### Other toxicities

Patient 16 stopped imatinib treatment because of diarrhea grade 3 that was then treated with loperamide and i.v. fluids. Patient 17 discontinued imatinib due to leukopenia and nausea in an adjuvant setting. Upon relapse, sunitinib therapy was started but withdrawn after 3 weeks due to hypertension, thrombopenia and consecutive intracerebral bleeding. Nilotinib was then well tolerated until progression. Patient 18 received imatinib in an adjuvant setting without toxicity. Upon local relapse imatinib was re-started and after 16 months on treatment the patient experienced severe esophagitis. A suspected viral infection was excluded. Symptoms improved after an imatinib treatment break. Nilotinib led to a transient arterial hypertension grade 2.

### Selected case presentations

**Figure 1.**
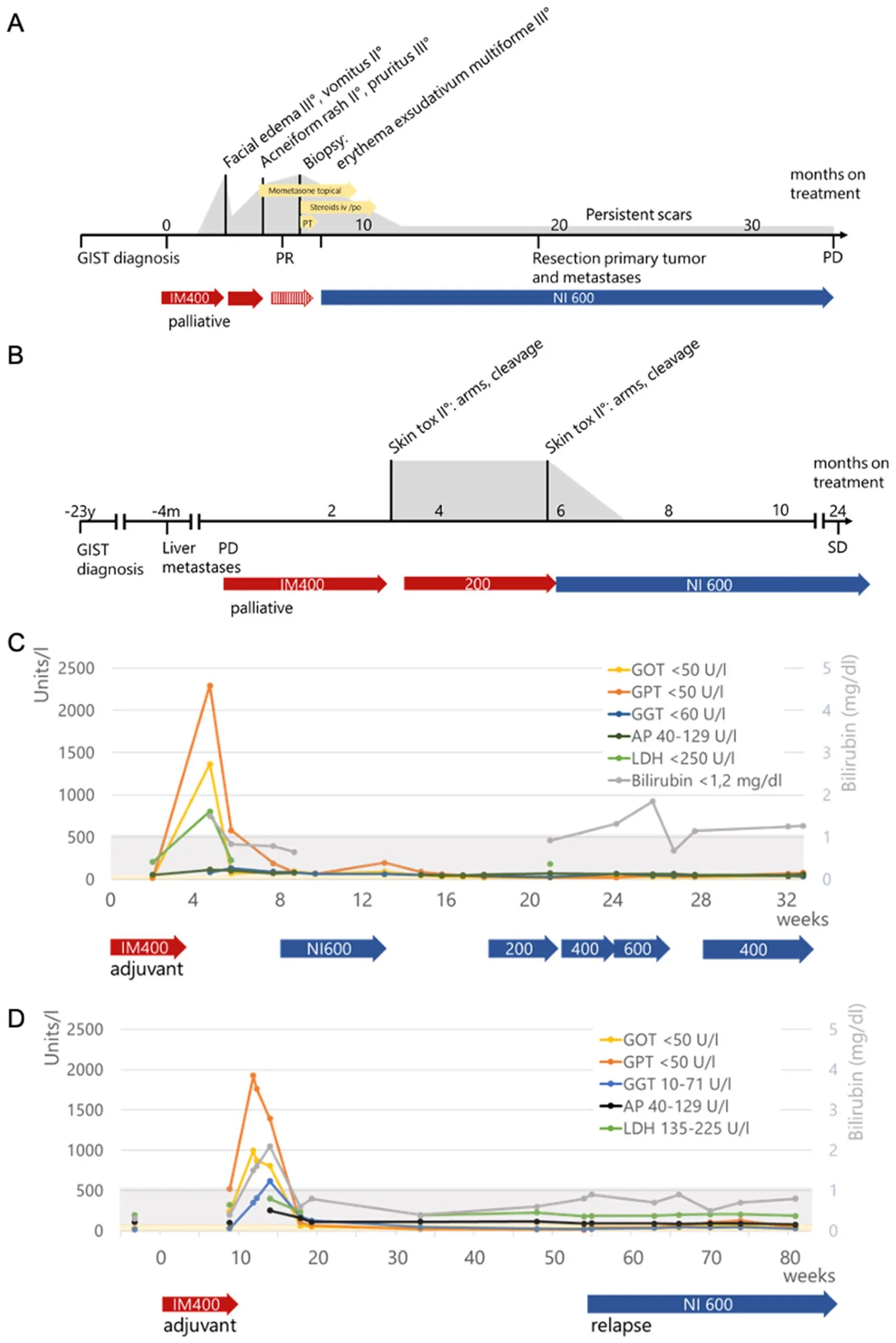
Case presentations of patients 5, 2, 13, and 14 (A–D, respectively). red arrows: Imatinib (IM) treatment and daily dose, blue arrows: nilotinib (NI) treatment and cumulative daily dose. (A and B) Skin toxicity. Orange arrows: medical treatment for toxicity. Gray shadows depict severity and course of toxicities. (C and D) Liver toxicity (light gray area depicts the normal level of bilirubin (<1,2mg/dl), light yellow depicts the normal GOT/GPT levels (<50U/l)).

We report more detailed on four patients with the most common toxicities (skin and liver):

Patient 5, a 65-year-old female patient with KIT Exon 11-mutant gastric GIST and synchronous peritoneal metastasis developed severe skin toxicity following 3 months of imatinib 400 mg qd. Treatment pause improved symptoms with early recurrence and aggravation after restart. The patient was hospitalized and whole-body erythema exsudativum multiforme was diagnosed by biopsy. Imatinib was discontinued and symptoms improved with systemic steroid therapy and phototherapy (PT). Treatment was changed to nilotinib and no additional skin toxicity occurred (Figure 1A).

Patient 2 was a 28-year-old female patient with ileal GIST. 23 years after resection, liver metastasis was diagnosed. Following progression, imatinib treatment was started, leading to grade II skin toxicity at arms and cleavage. Imatinib was paused for one week and restarted at a dose of 200 mg. After symptoms persisted, treatment was changed to nilotinib full dose. Skin toxicity resolved and did not recur during nilotinib treatment. A stable disease lasting for > 37 months was achieved (Figure 1B).

Patient 12, a 52-year-old man had a small intestinal GIST. Following complete resection and high risk of relapse, adjuvant imatinib treatment was started. Due to liver toxicity IV° imatinib was stopped after 3 weeks of treatment and prednisone was administered. Viral hepatitis serology was negative. Auto-immune hepatitis was also excluded. As salvage treatment, nilotinib was started after 5 weeks of treatment break. GPT increased II° and nilotinib was paused once after 5 weeks of treatment, re-escalation was tolerated well up to 400 mg, 600 mg led to bilirubin increase G2. Liver and peritoneal metastasis was diagnosed after 46 months of treatment. Metastases were resected. Treatment was continued with NI 400 mg qd after resection (Figure 1C).

Patient 14 was a 34-year-old man with small intestinal GIST. Tumor was resected and the relapse risk was classified as high. Imatinib 400 mg was started and after 6 weeks bilirubin and transaminases increased G2 and G4, respectively. Treatment was discontinued for approx. 12 months until peritoneal metastases were diagnosed. Then nilotinib was started at a dose of 600 mg qd. Nilotinib was then well tolerated with skin eczema I° as the only adverse event. At last follow-up the patient was on treatment for 14 months in partial remission (Figure 1D).

### Nilotinib was effective after imatinib treatment

**Figure 2.**
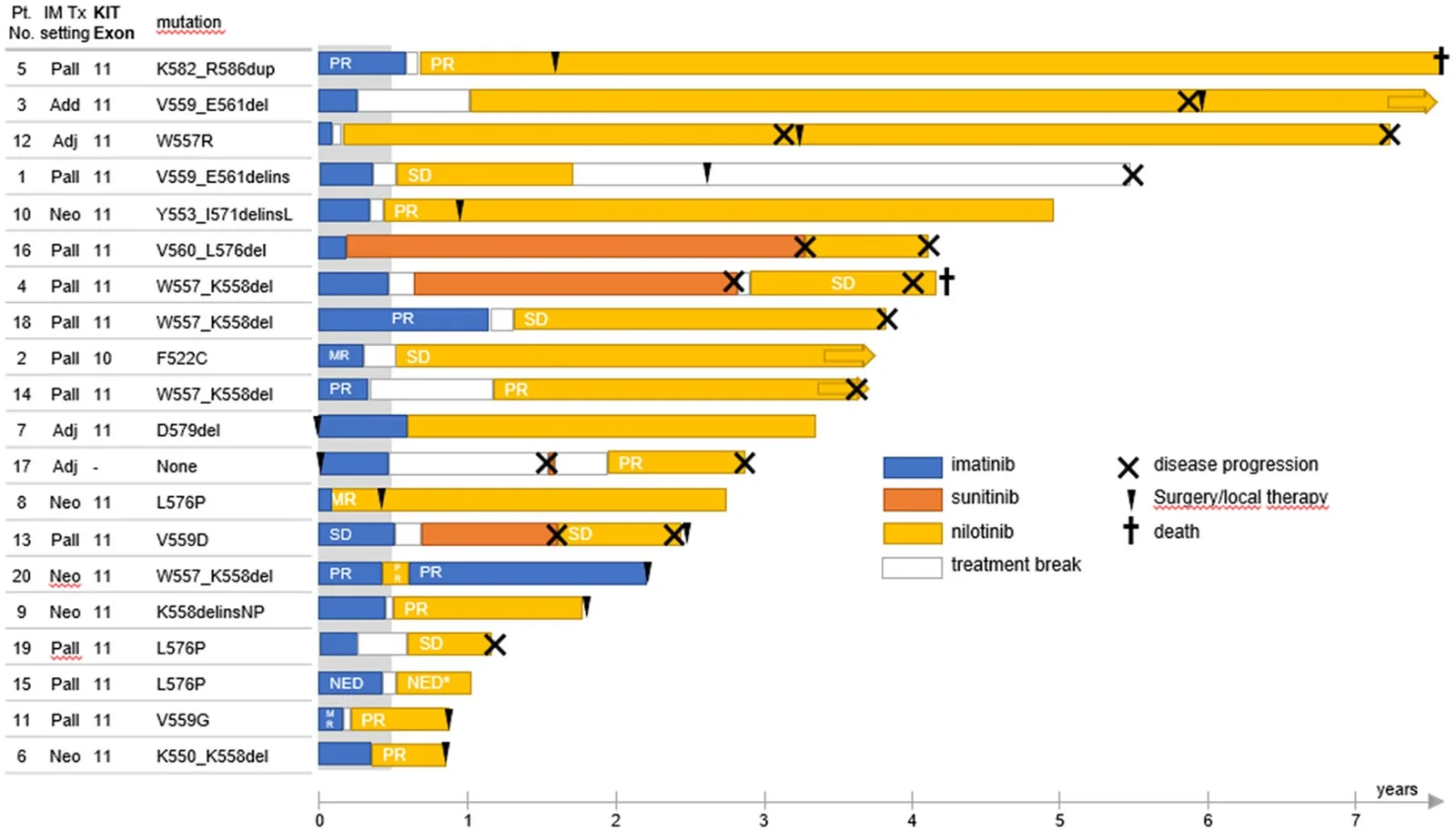
Swimmer plot from start of imatinib treatment until end of nilotinib treatment or end of follow-up. Arrows represent ongoing treatment at last follow-up. *Treatment withdrawn at the end of financial reimbursement. None: no KIT/PDGFRA mutation.

The median treatment duration was 65.5 weeks (range 9-386 weeks). The median duration of nilotinib response was evaluable in 7 patients and was 31 (6-152) weeks within the palliative treatment setting. Eight patients received surgery during nilotinib treatment either due to localized progression (*n* = 1) or upon response (neoadjuvant: *n* = 4, palliative *n* = 3) to reduce tumor load. Three patients were still on treatment without progression. The best radiologic response achieved was partial remission in 8, mixed response in 1, stable disease in 7, and NED after surgery in 4 patients, leading to an overall response rate of 50% (8/16) and a clinical benefit rate (SD + PR) of 93,8% (15/16 evaluable patients, Table 3). In one case (Pt. 15) nilotinib had to be withdrawn because treatment was covered by their health insurance for 6 months only. Patient 20 decided to switch back to imatinib due to hair loss during nilotinib treatment. Four patients received sunitinib after imatinib toxicity. In 3 cases (Pts. 4, 13, and 16) nilotinib was administered after sunitinib progression. In Pt. 17 severe sunitinib toxicity occurred, and treatment was withdrawn after 10 days. In this case the patient experienced imatinib and sunitinib toxicity. Nilotinib was then well tolerated and given until progression (11 months) (Figure 2).

### A de novo exon 9 mutation causes nilotinib resistance

In patient 12, a progressing hepatic lesion was removed surgically. Mutational analysis revealed a secondary mutation in *KIT* exon 9 (S476I) as a putative previously undescribed mechanism of nilotinib resistance. In Patient 13 who received nilotinib after sunitinib progression a new metastasis was surgically removed after 9 months of treatment, bearing an Exon 17 resistance mutation (p.K826_N828del).

### GIST cell line panel reveals nilotinib activity profile

**Figure 3.**
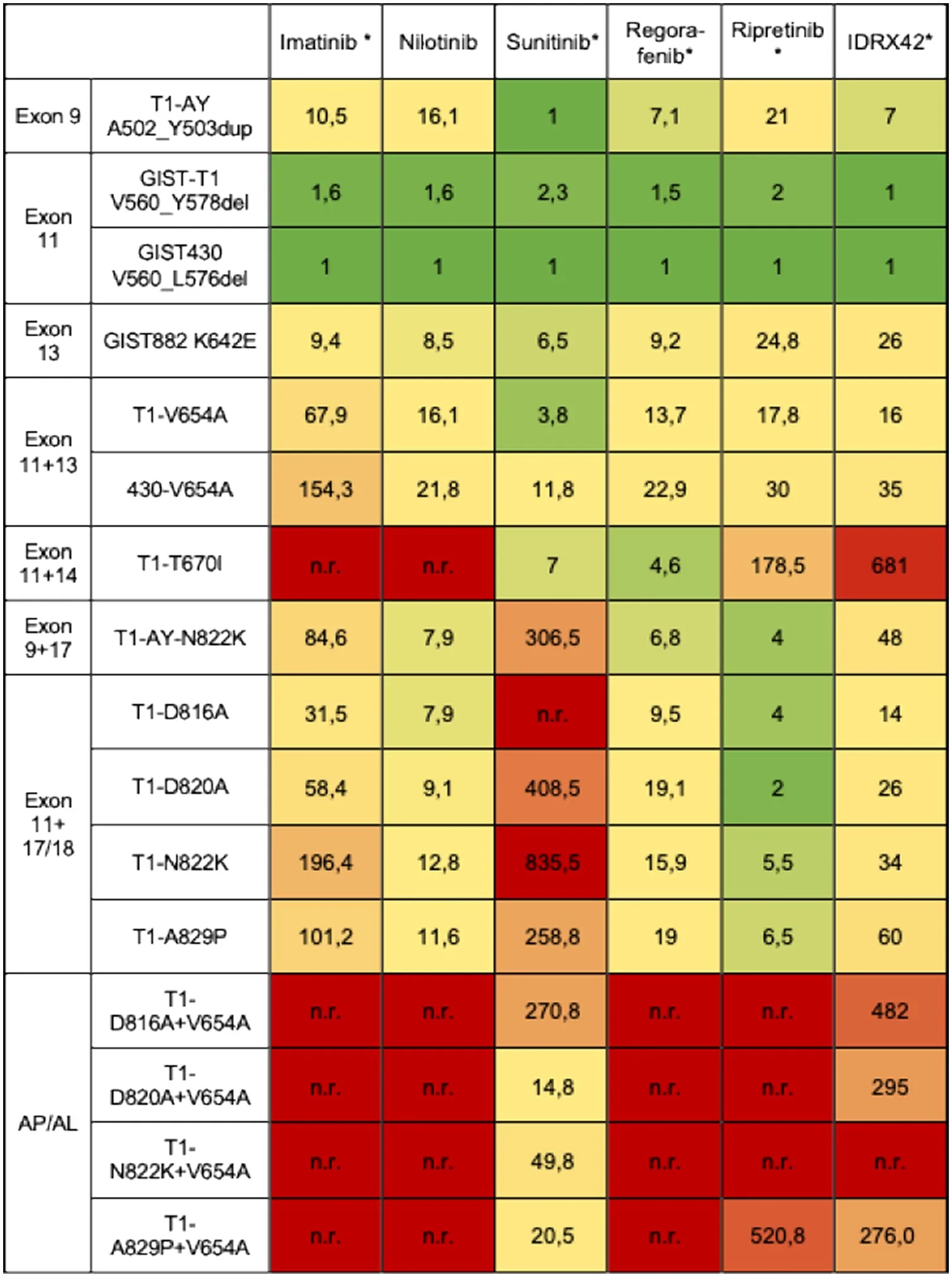
GR50 quotients: X-fold increase in doses needed for 50% growth rate inhibition (GR50) compared to Exon-11-mutant GIST430. Color code from green (1-fold) to red (GR50 n.r.= not reached, calculation not possible). Exact values are provided in each cell. *Published data^18^ posted for comparison.

We used an established GIST cell line panel including GIST standard cell lines as well as isogenic cell lines genetically engineered to bear common resistance mutations.^18^ GR50 values were normalized on GIST430, an Exon-11-mutant cell line with known nilotinib-sensitivity. Both Exon-11-mutant cell lines showed similar sensitivities toward all inhibitors. Sensitivity was much lower in the exon-9-mutant cell line (10.5-fold increase in GR50). All cell lines harboring additional mutations within the kinase domain were resistant (EC50 at least 9-fold compared to GIST430, Figure 3). GIST cell lines carrying primary Kit Exon 17/18 mutations are not established yet.

### Structure-based modeling of KIT in complex with nilotinib

**Figure 4.**
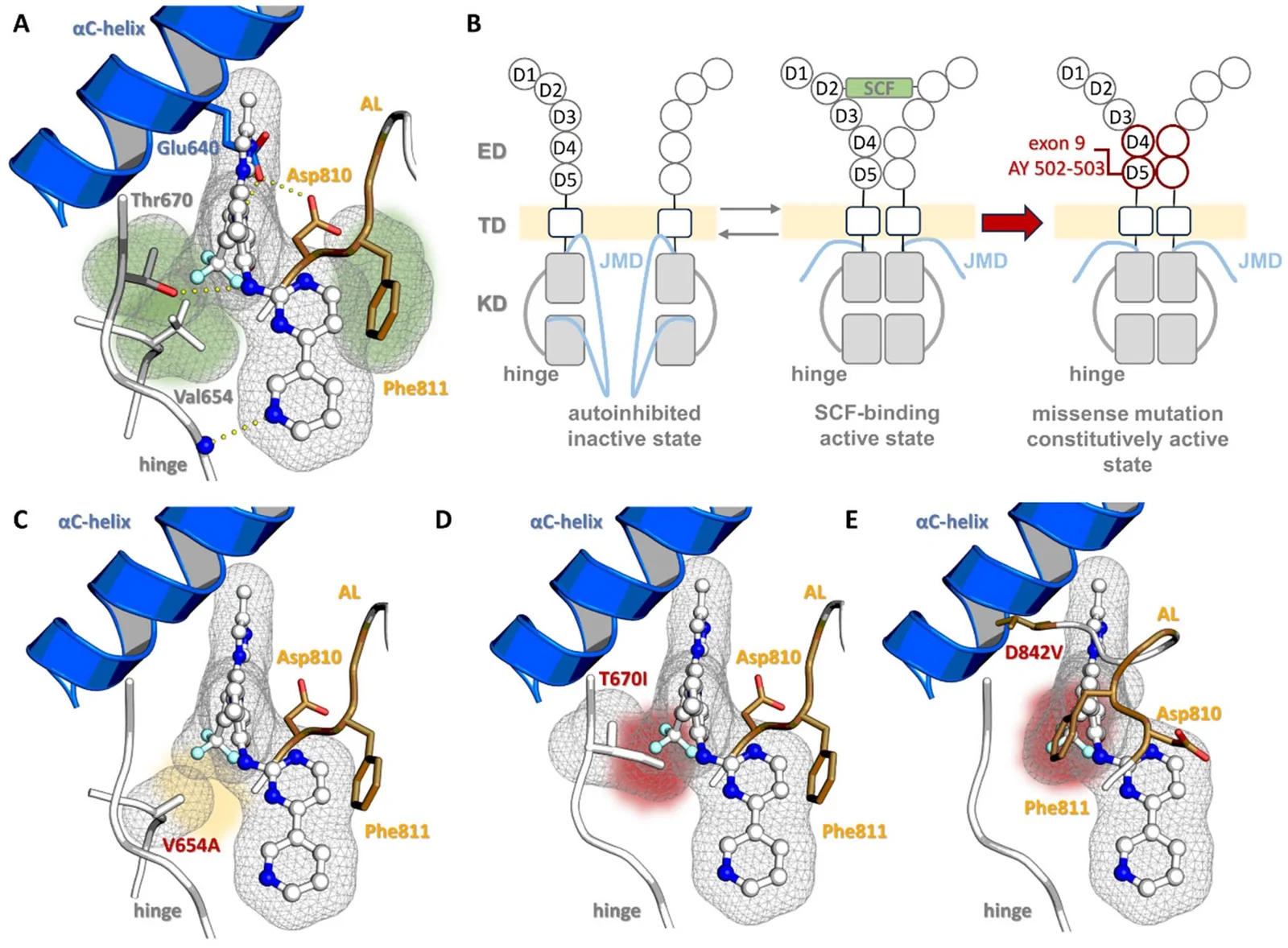
Anticipated structural consequences of KIT mutations on nilotinib binding and resistance mechanisms. (A) Modeled binding mode of nilotinib to KIT-WT (based on PDB ID 1T46 in superimposition with ABL-nilotinib, PDB ID 3CS9), showing stabilization of the inactive (DFG-out) conformation and interactions with Exon 13 Val654 and Exon 14 Thr670. Schematic representation of KIT domain architecture including extracellular domain (ED), transmembrane domain (TD), juxtamembrane domain (JMD), and kinase domain (KD), as well as binding of stem cell factor (SCF). (B) Schematic representation of KIT exon 9 mutations promoting ligand-independent dimerization and constitutive activation, shifting the kinase toward the active conformation incompatible with nilotinib binding. (C) Exon 13 V654A mutation results in loss of hydrophobic interactions with nilotinib (highlighted). (D) Exon 14 T670I gatekeeper mutation abolishes hydrogen bond formation and introduces steric hindrance to nilotinib binding (highlighted). (E) Activation loop (AL) mutations in Exons 17/18 favor the active (DFG-in) conformation, inducing steric clash with Phe811 and preventing nilotinib binding (in red).

To understand the structural mechanisms of nilotinib resistance, we used structure-based modeling. By superimposing the KIT structure on the nilotinib-bound ABL1 kinase structure^22^ (Figure 4A and Figure S2), nilotinib, as a type II inhibitor, seems to stabilize the inactive conformation of KIT. Nilotinib binding is accommodated without steric hindrance in the inactive conformation. Conversely, in the active conformation, Phe811 would sterically clash with nilotinib, suggesting that nilotinib binding is only compatible with the inactive conformation of the kinase. KIT exon 9 mutations (most common A502_Y503dup) have been shown to constitutively activate the oncogenic receptor (Figure 4B).^28^ Of note, nilotinib was predicted to form hydrophobic interactions with Exon 13 Val654 (Val299 in ABL) and hydrogen bonds with the gatekeeper Exon 14 Thr670 (Thr315 in ABL). The Exon 13 V654A mutation replaces Val with Ala, reducing hydrophobic interactions due to the smaller side chain (Figure 4C). Similarly, the Exon 14 T670I gatekeeper mutation introduces a bulkier, more lipophilic Ile that disrupts critical hydrogen bonds and creates a steric clash with nilotinib (Figure 4D). Furthermore, mutations in the activation loop (Exons 17/18, AL) shift the entire kinase domain into its active conformation. This shift reinstates steric hindrance (similar to that seen with exon 9 mutations) and prevents nilotinib binding (Figure 4E). The cumulative effects of these mutations account for the diminished efficacy observed with tertiary mutations affecting both the ATP-binding region and the activation loop. More detailed structural information is provided in the supplemental information.

## Discussion

Patients with metastatic gastrointestinal stromal tumors (GIST) require lifelong therapy with tyrosine kinase inhibitors (TKIs).^3^ Imatinib has been uniquely well tolerable compared to other drugs approved in GIST which allows patients to maintain role functioning and quality of life. Side effects of sunitinib and regorafenib, the second- and third-line treatment options, were shown to have a stronger impact in quality of life.^6^^,^^7^ Additionally, effectiveness of these drugs has never been tested in a non-imatinib-resistant first-line setting.

For patients on treatment with imatinib, mild side effects are frequent, but most patients respond to dose modifications and/or supportive treatments such as skin ointments (including steroids), diuretics, anti-diarrheal drugs. Following long-term use, a risk of secondary malignancies, as well as cardiac toxicities are reported.^29^ Albeit rare, patients can develop severe side effects requiring permanent discontinuation of imatinib treatment. Currently, the only approved option is switching to second-line sunitinib, which is associated with a much less favorable toxicity profile.^6^^,^^7^

Nilotinib was developed and is approved for the treatment of chronic myelogenous leukemia (CML). Given its KIT-inhibitory profile and the superiority to imatinib in CML, nilotinib was also tested in a first-line setting in GIST compared to imatinib (phase 3 ENESTg1 trial). This study was powered for superiority, and the overall result was negative. However, the study suggested equal effectivity in patients with KIT exon 11 mutations. The tolerability profile was comparable to imatinib.^13^ Additionally, nilotinib has demonstrated no significant cross-toxicity with imatinib in previous trials in CML.^30^^,^^31^ However, in patients treated with nilotinib cardiovascular events, QTc prolongations as well as fluid retention, pancreatitis and elevated blood glucose levels were more common compared to imatinib treatment.^32^ We are the first to publish a series of 20 GIST patients whose therapy was switched to nilotinib following severe or intolerable imatinib toxicity, providing a rationale for the use of nilotinib in this setting.

In our cohort 6-10% of patients quit imatinib treatment due to side effects with a higher incidence within the adjuvant setting. In this cohort of nilotinib-treated patients the reasons for discontinuation were skin and liver toxicity, cardiac disorders as well as diarrhea. Our cohort included more women than men (14 vs 6) well in line with the finding that women exhibited higher plasma levels for imatinib at the standardized dose of 400 mg/d.^33^ The frequency of toxicities leading to imatinib discontinuation differed from the numbers reported for the CML cohort as liver toxicity and skin toxicity were more common within our cohort (liver tox: 4/20 vs 4/75 cases; skin tox 11/20 vs 33/74 cases). The CML cohort had more fluid retention, diarrhea and myalgia/arthralgia as a reason for discontinuation.^30^ However, the types of toxicities observed were similar. Of note, grade 3 and 4 toxicities reported within clinical trials of imatinib in GIST were similar to those reported in our cohort^11^^,^^12^^,^^34^ (Table 3). The strong visibility of skin toxicity and the risk of drug-induced severe liver injury might explain why these were the main reasons for imatinib withdrawal. In this cohort, treatment break and the application of local or systemic steroids were the treatments chosen by the treating physicians. The exclusion of other underlying diseases is strongly encouraged.

We did not observe relevant cross toxicities in our cohort well in line with reported safety data of CML patients. This can be explained by structural differences within the nilotinib and imatinib molecules that cause differences in acidity and solubility. This also leads to different elimination routes and drug distribution.^30^^,^^31^ We conclude that nilotinib is a suitable treatment option in patients with GIST and primary exon 11 mutations who experience severe imatinib toxicity.

Nilotinib was tested in clinical trials within 2 scenarios. The ENESTg1 trial evaluated nilotinib vs imatinib in a first-line setting. Nilotinib was inferior regarding PFS and OS. The only subset with similar PFS outcomes was the exon-11-positive cohort (mPFS imatinib 32.2 months, nilotinib >30 months) while no benefit was shown for exon 9-mutant patients (mPFS imatinib >30 months vs nilotinib: 3 months).^13^ In our cohort, nilotinib seemed to be effective in Exon-11-mutant patients without progression on imatinib treatment. The low number of cases and multimodal treatment strategies preclude statistical effectivity analyses.

Phase 2 and phase 3 clinical trials evaluating nilotinib in late line scenarios had an mPFS of 2-3.7 months, indicating lacking sensitivity in imatinib-resistant GIST.^14–16^ Patients that were treated with nilotinib following sunitinib progression had a treatment duration that was 6-12 months (3 patients), which was shorter compared to the previous sunitinib duration of response.

Nilotinib has been initially developed using a rational drug design approach based on imatinib in order to improve its binding affinity for BCR::ABL1. However, the GIST cell line panel (Figure 3) reveals that cell lines harboring additional KIT mutations (eg, e9 + 17, e11 + 13, e11 + 17/18) were resistant to nilotinib. Interestingly though, nilotinib shows an enhanced potency over imatinib (approximately 4-15-fold) in these cell lines, which can be attributed to structural differences between the 2 drugs. Nilotinib’s trifluoromethyl group enables it to penetrate a hydrophobic pocket within KIT, increasing drug–protein interactions, similar to its effect on ABL. Despite of the improved interaction profile, the potency of nilotinib is not sufficient to counter the steric clashes or disrupted interactions caused by secondary mutations, rendering these cell lines resistant to nilotinib. Thus, nilotinib does not reach the therapeutic window to overcome secondary mutations in KIT.

One patient within our cohort had a secondary exon 9 mutation S476I found within a progressing liver metastasis. In other publications, the effectiveness of nilotinib has been compared with other TKIs. Inhibitory concentrations (IC50s) above 250 nM were described as sensitive in these studies. To see if the results of our panel are comparable to previously published data, we identified one cell line (GIST882) that was used by the other group and within our experiments. This cell line, carrying a primary exon 13 mutation (K642E) had similar inhibitory concentrations (IC50 160 nM,^22^ EC50 144 nM in our model). Additionally, the inference of greater efficacy was drawn from the lower inhibitory dosage compared to imatinib.^22^ GIST cell line models as well as clinical data for primary KIT Exon 17 mutations do not exist. However, other literature suggests that the cellular uptake of nilotinib differs from that of other TKIs, implying that direct comparisons of dosages may not be appropriate.^31^ Structural analyses explained the lack in efficacy in exon-9-mutant GIST and GIST harboring imatinib resistance mutations.

In conclusion, our data suggest that the switch to nilotinib after imatinib-related toxicities should be preferred over sunitinib for patients due to several factors. Nilotinib offers a more favorable adverse event profile compared to sunitinib and regorafenib. A comparable financial toxicity post-patent expiration can simplify the application for cost coverage by insurances. Additionally, given sunitinib’s potent AP-mutation-inhibitory activity,^18^ it is advisable to reserve its use for situations of drug resistance, as its unnecessary use may potentially promote the emergence of resistance-associated mutations, such as activation loop (AL) mutations.

## Conclusion

Within a first-line setting before progression, nilotinib is a tolerable and efficient salvage treatment option for patients with exon 11-mutant GIST experiencing severe imatinib toxicity. As nilotinib is not approved for GIST treatment, a written consent of the insurances should be obtained and adverse events should be monitored closely. Nilotinib does not have a rationale in patients with exon-9-mutant GIST and GIST with primary exon 11 and additional secondary resistance mutations in KIT.

## Supplementary Material

oyag345_Supplementary_Data

## Data Availability

The data underlying this article will be shared on reasonable request to the corresponding author.

## References

[1] Hirota S , Nishida T, Isozaki K, et al Gain-of-function mutation at the extracellular domain of KIT in gastrointestinal stromal tumours. J. Pathol. 2001;193:505-510. 10.1002/1096-9896(2000)9999:9999<::AID-PATH818>3.0.CO;2-E 11276010

[2] Hirota S , Ohashi A, Nishida T, et al Gain-of-function mutations of platelet-derived growth factor receptor alpha gene in gastrointestinal stromal tumors. Gastroenterology. 2003;125:660-667. 10.1016/s0016-5085(03)01046-1 12949711

[3] Casali PG , Blay JY, Abecassis N, et al ESMO Guidelines Committee, EURACAN and GENTURIS. Electronic address: clinicalguidelines@esmo.org. Gastrointestinal stromal tumours: ESMO-EURACAN-GENTURIS Clinical Practice Guidelines for diagnosis, treatment and follow-up. Ann Oncol. 2022;33:20-33. 10.1016/j.annonc.2021.09.005 34560242

[4] Søreide K , Sandvik OM, Søreide JA, Giljaca V, Jureckova A, Bulusu VR. Global epidemiology of gastrointestinal stromal tumours (GIST): a systematic review of population-based cohort studies. Cancer Epidemiol. 2016;40:39-46. 10.1016/j.canep.2015.10.031 26618334

[5] Corless CL , Barnett CM, Heinrich MC. Gastrointestinal stromal tumours: origin and molecular oncology. Nat Rev Cancer. 2011;11:865-878. 10.1038/nrc3143 22089421

[6] Demetri GD , Reichardt P, Kang Y-K, et al GRID Study Investigators. Efficacy and safety of regorafenib for advanced gastrointestinal stromal tumours after failure of imatinib and sunitinib (GRID): an international, multicentre, randomised, placebo-controlled, phase 3 trial. Lancet. 2013;381:295-302. 10.1016/S0140-6736(12)61857-1 PMC3819942 23177515

[7] Demetri GD , van Oosterom AT, Garrett CR, et al Efficacy and safety of sunitinib in patients with advanced gastrointestinal stromal tumour after failure of imatinib: a randomised controlled trial. Lancet. 2006;368:1329-1338. 10.1016/S0140-6736(06)69446-4 17046465

[8] Ferguson RJ , Manculich J, Chang H, et al Self-reported cognitive impairments and quality of life in patients with gastrointestinal stromal tumor: results of a multinational survey. Cancer. 2022;128:4017-4026. 10.1002/cncr.34469 PMC9633548 36125989

[9] Blay J-Y , Serrano C, Heinrich MC, et al Ripretinib in patients with advanced gastrointestinal stromal tumours (INVICTUS): a double-blind, randomised, placebo-controlled, phase 3 trial. Lancet Oncol. 2020;21:923-934. 10.1016/S1470-2045(20)30168-6 PMC8383051 32511981

[10] Schöffski P , George S, Heinrich MC, et al Patient-reported outcomes in individuals with advanced gastrointestinal stromal tumor treated with ripretinib in the fourth-line setting: analysis from the phase 3 INVICTUS trial. BMC Cancer. 2022;22:1302. 10.1186/s12885-022-10379-9 PMC9746146 36514034

[11] Verweij J , Casali PG, Zalcberg J, et al Progression-free survival in gastrointestinal stromal tumours with high-dose imatinib: randomised trial. Lancet. 2004;364:1127-1134. 10.1016/S0140-6736(04)17098-0 15451219

[12] Blanke CD , Rankin C, Demetri GD, et al Phase III randomized, intergroup trial assessing imatinib mesylate at two dose levels in patients with unresectable or metastatic gastrointestinal stromal tumors expressing the kit receptor tyrosine kinase: S0033. J Clin Oncol. 2008;26:626-632. 10.1200/JCO.2007.13.4452 18235122

[13] Blay J-Y , Shen L, Kang Y-K, et al Nilotinib versus imatinib as first-line therapy for patients with unresectable or metastatic gastrointestinal stromal tumours (ENESTg1): a randomised phase 3 trial. Lancet Oncol. 2015;16:550-560. 10.1016/S1470-2045(15)70105-1 PMC4521211 25882987

[14] Cauchi C , Somaiah N, Engstrom PF, et al Evaluation of nilotinib in advanced GIST previously treated with imatinib and sunitinib. Cancer Chemother Pharmacol. 2012;69:977-982. 10.1007/s00280-011-1785-7 PMC3313017 22119758

[15] Montemurro M , Schöffski P, Reichardt P, et al Nilotinib in the treatment of advanced gastrointestinal stromal tumours resistant to both imatinib and sunitinib. Eur J Cancer. 2009;45:2293-2297. 10.1016/j.ejca.2009.04.030 19467857

[16] Sawaki A , Nishida T, Doi T, et al Phase 2 study of nilotinib as third-line therapy for patients with gastrointestinal stromal tumor. Cancer. 2011;117:4633-4641. 10.1002/cncr.26120 21456006

[17] Reichardt P , Blay J-Y, Gelderblom H, et al Phase III study of nilotinib versus best supportive care with or without a TKI in patients with gastrointestinal stromal tumors resistant to or intolerant of imatinib and sunitinib. Ann Oncol. 2012;23:1680-1687. 10.1093/annonc/mdr598 22357255

[18] Mühlenberg T , Falkenhorst J, Schulz T, et al KIT ATP-binding pocket/activation loop mutations in GI stromal tumor: Emerging mechanisms of kinase inhibitor escape. J Clin Oncol. 2024;42:1439-1449. 10.1200/JCO.23.01197 PMC11095889 38408285

[19] Grunewald S , Klug LR, Mühlenberg T, et al Resistance to avapritinib in PDGFRA-driven GIST is caused by secondary mutations in the PDGFRA kinase domain. Cancer Discov. 2021;11:108-125. 10.1158/2159-8290.CD-20-0487 32972961

[20] Skehan P , Storeng R, Scudiero D, et al New colorimetric cytotoxicity assay for anticancer-drug screening. J Natl Cancer Inst. 1990;82:1107-1112. 10.1093/jnci/82.13.1107 2359136

[21] Hafner M , Niepel M, Chung M, Sorger PK. Growth rate inhibition metrics correct for confounders in measuring sensitivity to cancer drugs. Nat Methods. 2016;13:521-527. 10.1038/nmeth.3853 PMC4887336 27135972

[22] Weisberg E , Manley PW, Breitenstein W, et al Characterization of AMN107, a selective inhibitor of native and mutant Bcr-Abl. Cancer Cell. 2005;7:129-141. 10.1016/j.ccr.2005.01.007 15710326

[23] Cowan-Jacob SW , Fendrich G, Floersheimer A, et al Structural biology contributions to the discovery of drugs to treat chronic myelogenous leukaemia. Acta Crystallogr D Biol Crystallogr. 2007;63:80-93. 10.1107/s0907444906047287 PMC2483489 17164530

[24] Mol CD , Lim KB, Sridhar V, et al Structure of a c-kit product complex reveals the basis for kinase transactivation*. J Biol Chem. 2003;278:31461-31464. 10.1074/jbc.C300186200 12824176

[25] Mol CD , Dougan DR, Schneider TR, et al Structural basis for the autoinhibition and STI-571 inhibition of c-kit tyrosine kinase. J Biol Chem. 2004;279:31655-31663. 10.1074/jbc.M403319200 15123710

[26] Wagner AJ , Severson PL, Shields AF, et al Association of combination of conformation-specific KIT inhibitors with clinical benefit in patients with refractory gastrointestinal stromal tumors: a phase 1b/2a nonrandomized clinical trial. JAMA Oncol. 2021;7:1343-1350. 10.1001/jamaoncol.2021.2086 PMC8267845 34236401

[27] Wolber G , Langer T. LigandScout: 3-D pharmacophores derived from protein-bound ligands and their use as virtual screening filters. J Chem Inf Model. 2005;45:160-169. 10.1021/ci049885e 15667141

[28] Reshetnyak AV , Opatowsky Y, Boggon TJ, et al The strength and cooperativity of KIT ectodomain contacts determine normal ligand-dependent stimulation or oncogenic activation in cancer. Mol Cell. 2015;57:191-201. 10.1016/j.molcel.2014.11.021 PMC4764128 25544564

[29] Hochhaus A , Larson RA, Guilhot F, et al IRIS Investigators. Long-term outcomes of imatinib treatment for chronic myeloid leukemia. N Engl J Med. 2017;376:917-927. 10.1056/NEJMoa1609324 PMC5901965 28273028

[30] Cortes JE , Hochhaus A, Le Coutre PD, et al Minimal cross-intolerance with nilotinib in patients with chronic myeloid leukemia in chronic or accelerated phase who are intolerant to imatinib. Blood. 2011;117:5600-5606. 10.1182/blood-2010-11-318949 PMC4186645 21467546

[31] Manley PW , Stiefl N, Cowan-Jacob SW, et al Structural resemblances and comparisons of the relative pharmacological properties of imatinib and nilotinib. Bioorg Med Chem. 2010;18:6977-6986. 10.1016/j.bmc.2010.08.026 20817538

[32] Kantarjian HM , Hughes TP, Larson RA, et al Long-term outcomes with frontline nilotinib versus imatinib in newly diagnosed chronic myeloid leukemia in chronic phase: ENESTnd 10-year analysis. Leukemia. 2021;35:440-453. 10.1038/s41375-020-01111-2 PMC7862065 33414482

[33] Demetri GD , Wang Y, Wehrle E, et al Imatinib plasma levels are correlated with clinical benefit in patients with unresectable/metastatic gastrointestinal stromal tumors. J Clin Oncol. 2009;27:3141-3147. 10.1200/jco.2008.20.4818 19451435

[34] Demetri GD , von Mehren M, Blanke CD, et al Efficacy and safety of imatinib mesylate in advanced gastrointestinal stromal tumors. N Engl J Med. 2002;347:472-480. 10.1056/NEJMoa020461 12181401

