# Supplementary material for: Nilotinib revisited in GIST: salvage use in patients with severe imatinib toxicity
Source: Oncologist. 2026 Sep 3;31(10):oyag345. doi: 10.1093/oncolo/oyag345 (PMC13596059; doi:10.1093/oncolo/oyag345)
Supplement: Supplementary file 1 [file oyag345_supplementary_data.docx]

Supplemental Information

Supplemental Figure S1: Consort Diagram

Supplemental Figure S2: Pharmacophore model and interactions of (A) nilotinib (by superimposition of the KIT-imatinib complex (PDB ID 1T46) and the ABL1-nilotinib complex (PDB ID 3CS9)) and (B) imatinib in KIT (PDB ID 1T46); pharmacophore model of (C) nilotinib (PDB ID 3CS9) and (D) imatinib in ABL1 (PDB ID 2HYY).

Stucture-based modeling

## **Structure-based modeling of KIT in complex with nilotinib**

In the absence of crystallographic data for the KIT-nilotinib complex, we used structure-based modeling to elucidate the mechanisms underlying nilotinib resistance in gastrointestinal stromal tumors (GIST). By superimposing the KIT structure on the nilotinib-bound ABL kinase structure (28) (Figure 3 A and Supplemental Figure S2), our analysis suggests that nilotinib acts as a type II inhibitor, stabilizing the inactive conformation of KIT, analogous to its interaction with ABL. Specifically, nilotinib is predicted to form hydrophobic interactions with Val654 (corresponding to Val299 in ABL) and hydrogen bonds with the gatekeeper Thr670 (analogous to Thr315 in ABL). Together with the DFG motif amino acids Asp810-Phe811-Gly812 (corresponding to Asp381-Phe382-Gly383 in ABL), these residues are conserved between the two kinases. Nilotinib binding is accommodated without steric hindrance in the inactive (DFG-out) conformation. Conversely, in the active (DFG-in) conformation, Phe811 would sterically clash with nilotinib, suggesting that nilotinib binding is only compatible with the inactive conformation of the kinase. KIT exon 9 mutations have been shown to constitutively activate the oncogenic receptor. These mutations, which occur in the membrane-proximal Ig-like domain D5, such as AY 502-503 dup, enhance homotypic interactions, significantly increase dimerization affinity, and lead to ligand-independent activation (Figure 3 B) (29). This, in turn, affects the tyrosine kinase domain, shifting the kinase domain toward the active conformation and inducing the previously described steric clash with Phe811 in the DFG motif. Secondary resistance to nilotinib in KIT can be explained by distinct mutations. The V654A mutation replaces Val with Ala, reducing hydrophobic interactions due to the smaller side chain (Figure 3 C). Similarly, the T670I gatekeeper mutation introduces a bulkier, more lipophilic Ile that disrupts critical hydrogen bonds and creates a steric clash with nilotinib (Figure 3 D). Furthermore, mutations in the activation loop (AL) shift the DFG motif - and consequently the entire kinase domain - into its active conformation. This shift reinstates steric hindrance (similar to that seen with exon 9 mutations) and prevents nilotinib binding (Figure 3 E). The cumulative effects of these mutations account for the diminished efficacy observed with tertiary mutations affecting both the ATP-binding region and the activation loop.

Nilotinib has been initially developed using a rational drug design approach based on imatinib in order to improve its binding affinity for BCR-ABL. However, the GIST cell line panel (Table 4) reveals that cell lines harboring additional KIT mutations (e.g., e9+17, e11+13, e11+17/18) were resistant to nilotinib. Interestingly though, nilotinib shows an enhanced potency over imatinib (approx. 4-15 fold) in these cell lines, which can be attributed to structural differences between the two drugs. Nilotinib’s trifluoromethyl group enables it to penetrate a hydrophobic pocket within KIT, increasing drug-protein interactions, similar to its effect on ABL. Despite of the improved interaction profile, the potency of nilotinib is not sufficient to counter the steric clashes or disrupted interactions caused by secondary mutations, rendering these cell lines resistant to nilotinib. Thus, nilotinib does not reach the therapeutic window to overcome secondary mutations in KIT.
